# The Role of Vitamin D in COVID-19 and the Impact of Pandemic Restrictions on Vitamin D Blood Content

**DOI:** 10.3389/fphar.2022.836738

**Published:** 2022-02-21

**Authors:** Agata Tomaszewska, Agnieszka Rustecka, Agnieszka Lipińska-Opałka, Rafal P. Piprek, Małgorzata Kloc, Bolesław Kalicki, Jacek Z. Kubiak

**Affiliations:** ^1^ Pediatric, Nephrology, and Allergology Clinic, Military Institute of Medicine, Warsaw, Poland; ^2^ Department of Comparative Anatomy, Institute of Zoology and Biomedical Research, Jagiellonian University, Krakow, Poland; ^3^ The Houston Methodist Research Institute, Houston, TX, United States; ^4^ Department of Surgery, The Houston Methodist Hospital, Houston, TX, United States; ^5^ Department of Genetics, M.D. Anderson Cancer Center, The University of Texas, Houston, TX, United States; ^6^ Faculty of Medicine, CNRS, UMR 6290, Institute of Genetics and Development of Rennes, University Rennes, Rennes, France; ^7^ Laboratory of Molecular Oncology and Innovative Therapies, Military Institute of Medicine, Warsaw, Poland

**Keywords:** Vitamin D, COVID-19, SARS-CoV2, Cytokine storm, pandemic

## Abstract

Vitamin D is a hormone regulating the immune system and playing a pivotal role in responses to microbial infections. It regulates inflammatory processes by influencing the transcription of immune-response genes in macrophages, T cells, and dendritic cells. The proven role of vitamin D in many infectious diseases of the respiratory tract indicated that vitamin D should also play a role in SARS-CoV-2 infection. Vitamin D inhibits cytokine storm by switching the pro-inflammatory Th1 and Th17 to the anti-inflammatory Th2 and Treg response. Vitamin D is therefore expected to play a role in preventing, relieving symptoms, or treating SARS-CoV-2 infection symptoms, including severe pneumonia. There are several possible mechanisms by which vitamin D may reduce the risk of COVID-19 infection, such as induction of the transcription of cathelicidin and defensin. Also a nongenomic antiviral action of vitamin D and lumisterol, the molecule closely related to vitamin D, was reported. Despite this enormous progress, currently, there is still insufficient scientific evidence to support the claim that vitamin D supplementation may help treat COVID-19 infection. The pandemic restrictions were also shown to impact vitamin D uptake by limiting exposure to sunlight.

## Introduction

Vitamin D (25 (OH)2 D) is a hormone that controls the absorption and homeostasis of calcium, magnesium, and phosphate, regulating numerous aspects of human health, including immune and inflammatory responses to microbial infections. It is a fat-soluble secosteroid, thus the steroid with a “broken” ring (Holick, 2004; Holick, 2006).

The regulatory effect of vitamin D is systemic. Deficiencies of this vitamin are associated with an increased risk of various diseases, including respiratory system infections, diabetes, neuromuscular disorders, or cancer (Christakos et al., 2016). It can affect the cardiovascular system, reducing the risk of cardiovascular diseases (Zittermann and Prokop, 2014). It has also been proven that low vitamin D concentration are associated with the development of hypertension. Pathomechanism is related to the regulation of the renin-angiotensin-aldosterone system (RAA). Under normal conditions, the optimal concentration of vitamin D inhibits the activity of the RAA system by lowering the expression of the renin gene and the angiotensin 1 receptor (Mandarino et al., 2015). Reducing renin synthesis and inhibiting the activity of the RAA system enables the control of circulating blood volume and prevents the development of arterial hypertension.

Despite the proven positive impact of vitamin D on several aspects of health, its deficiencies are still common. According to epidemiological studies, vitamin D deficiency in the European population may cover 50–70% of the society, and in Poland, it may even be around 90% (Hilger et al., 2014; Rusińska et al., 2018).

The widespread deficiency of vitamin D in Polish society prompted experts to develop recommendations for its supplementation (Table 1) (Rusińska et al., 2018). Calcifediol - 25 (OH) D is used in patients with disorders of hepatic metabolism of vitamin D (including cholestasis, chronic treatment with glucocorticosteroids, or anticonvulsants). In turn, in the case of renal vitamin D metabolism disorders and diseases with a decrease in the activity of 1-alpha-hydroxylase (including chronic kidney disease, nephrotic syndrome, hypophosphatemic rickets), active metabolites and vitamin D analogs such as alfacalcidol calcitriol should be used.

**TABLE 1:** Recommended doses of vitamin D in the general population and in risk groups.

Group	The recommended dose of vitamin D
Newborns and infants	
Born <32 weeks pregnant	800 IU/day from the first days of life
Born 32–36 weeks pregnant	400 IU/day from the first days of life
0–6 months	400 IU/day from the first days of life
6–12 months	400–600 IU/day depending on the daily amount of vitamin D taken with food
Children 1–10 years	600–1000 IU/day
Youth 11–18 years	800–2000 IU/day
Adults 19–65 lat	800–2000 IU/day
Seniors 65–75 lat	800–2000 IU/day
Seniors >75 lat	2000–4,000 IU/day
People with dark complexion	800–2000 IU/day
Women during pregnancy and lactation	
During procreation and planning a pregnancy	800–2000 IU/day
During pregnancy	1500–2000 IU/day
Obese people	
Children and adolescent patients	1200–2000 IU/day
Adults	1600–4000 IU/day
People working at night	1000–2000 IU/day

### Molecular Action of Vitamin D in Immune Cells

The active metabolite of vitamin D, which is 1, 25 dihydroxy vitamin D (1,25 (OH)2D3), circulates in the blood and fulfills its hormone regulatory function through the binding to the specific receptor (Vitamin D Receptor; VDR). The latter is also called NR1I1 (Nuclear Receptor subfamily 1, group I, member 1). Vitamin D, together with its nuclear receptor, acts as a transcription factor, thus regulate processes *via* genomic action (Kongsbak et al., 2013; Bikle, 2016). The 1,25 (OH)2 D/VDR complex heterodimerizes with the retinoic-X receptor (RXR), which triggers their nuclear translocation allowing them to bind to the vitamin D response elements (VDREs) on DNA. This binding provokes the dissociation of repressors and allows to recruit co-factors, altogether regulating transcription of more than 900 genes (Moore et al., 2006). VDR is highly expressed in immune cells such as macrophages, dendritic cells, and T cells. Thus, not surprisingly, numerous target genes of vitamin D are responsible for immune response, immune modulation, inflammatory reactions, and responses to microbial infections (Kongsbak et al., 2013). The well-known targets of vitamin D-dependent transcriptional regulation are the cathelicidin and defensin genes coding for anti-microbial peptides inhibiting viral replication and promoting chemotaxis of macrophages and other immune cells to the sites of inflammation (Fiske et al., 2019).

An alternative pathway for noncalcemic 20-hydroxy- and 20,23-dihydroxyvitamin D synthesis involving retinoic acid receptor-related orphan receptors α and γ, namely RORα and RORγ, discovered in human skin cells were also described (Slominski et al., 2014). They are biologically active, and they were identified as major regulators of helper T (Th)17 cell differentiation, and thus play a pivotal role in the innate immune system functions and autoimmune disorders (Kojima et al., 2015). It is known that vitamin D and its biologically active derivatives play a regulatory role in the immune system impacting, among others, also the allergic reactions (Lipińska-Opałka et al., 2021). The presence of alternative pathways may explain the pleiotropic and so diverse activities of vitamin D, which were previously assigned solely to 1,25(OH)2D3 and VDR (Slominski et al., 2017).

Besides, the described above genomic action, vitamin D and closely related molecules, like lumisterol, participate in cellular physiology *via* non-genomic activities. It was postulated that they may exhibit potent activity against SARS-CoV-2 (Mok et al., 2020). Recently, Qayyum et al. (2021) have shown that this non-genomic action of vitamin D and lumisterol consist of active inhibition of SARS-CoV-2 replication machinery. The main viral protease M^pro^ activity was reduced by 10–19%, while viral RNA-dependent RNA polymerase RdRP activity was reduced by 50–60% (ibid.). Thus, the inhibitory action of vitamin D and lumisterol may play may play a highly significant role in active fight with SARS-CoV-2 infection and thus diminish the severity of COVID-19 progression in patients. Moreover, a structure-based virtual screening was used in this study to predict analogs that could bind to these two viral proteins followed by enzyme inhibition studies which confirmed these interactions. These exciting results provide strong support for the ability of D3, L3, and 7-DHC hydroxy-metabolites to reduce the viral load in infected cells or in the blood stream. Further molecular studies of vitamin D, similar steroids and their derivatives in COVID-19 patients may shed more light on their role in SARS-CoV-2 infection.

### Vitamin D and COVID-19

The SARS-CoV2 virus pandemic has affected many aspects of everyday life, from changing lifestyle behaviors to changing eating habits. The available studies show that in Poland, during the first months of the pandemic, 43% of adults decreased their physical activity, 39% - extended the time spent watching TV, and 34% - increased food consumption (Górnicka et al., 2020). Such changes are negative public health indicators because they are a prelude to the development of overweight and obesity. Reports showed that during the pandemic from spring to autumn 2020, 28% of Poles aged 20 or more (28% of men and 29% of women) experienced weight gain. Women aged 45–64 (36%) and men aged 20–44 (30%) gained weight most often (Wojtyniak and Goryński, 2020).

The food consumed by society and the micronutrients it contains play a key role in regulating the function of the human immune system. Among them, the special immunomodulatory role is attributed to vitamin D. Studies show that patients with vitamin D deficiency are more likely to develop severe complications of lower respiratory tract infections, show a 117% higher risk of requiring oxygen therapy, and a 217% higher risk of requiring mechanical ventilation than patients with correct concentration values of 25 (OH) D (Cebey-Lopez et al., 2015). The role of vitamin D is also well documented in reducing the severity and incidence of respiratory infections. There is a linear relationship between vitamin D concentration and the cumulative incidence of infection (Bergman et al., 2015). Infection frequency increases significantly with 25 (OH) D values < 30 ng/ml. (Monlezum et al., 2015).

The proven role of vitamin D in many infectious diseases of the respiratory tract indicated that vitamin D should also play a role in SARS-CoV-2 infection. One of the mechanisms of damage to lung tissue by SARS-CoV-2 is the increased production of Th1 pro-inflammatory cytokines (resulting in so-called cytokine storm), which leads to the development of acute respiratory failure (ARDS) (Lai et al., 2020). Vitamin D inhibits cytokine storm by switching the pro-inflammatory Th1 and Th17 to the anti-inflammatory Th2 and Treg response (Gorman et al., 2012).

The data mentioned above suggest that vitamin D should play a role in preventing, relieving symptoms, or treating SARS-CoV-2 infection symptoms, including severe pneumonia.

### Vitamin D in the Prevention of SARS-CoV2 Infections

The endocytic entry of the SARS-CoV-2 virus to the pneumocytes occurs after glycoprotein S (the viral Spike protein) binds to the angiotensin-2 receptor (ACE2) (Hoffman et al., 2020). Early response of the immune system to eliminate the virus at this stage is crucial in reducing the progression of the disease.

A retrospective study by D'Avolio et al. (2020) showed a clear correlation between vitamin D concentration in the blood and the risk of COVID-19. This study was conducted in March-April 2020 on 107 patients with symptoms of acute respiratory infection (27 with confirmed SARS-CoV2 infection, 80 with negative results). The control group consisted of 1,377 healthy patients whose vitamin D blood concentration was assessed between March and april 2019. It has been proven that patients with COVID-19 had statistically significantly lower concentrations of vitamin D as compared to patients with negative results for SARS-CoV-2 and healthy patients from 2019 (11.1 ng/ml *vs*. 24.6 ng/ml *vs*. 24.6 ng/ml, respectively). However, there was no difference in vitamin D content between the group with negative PCR tests for SARS-CoV-2 in 2020 and the group from 2019. Similar observations were made by Kaufman et al. (2020). The analysis was carried out on a group of 191,779 patients who tested positive for SARS-CoV-2 infection. There was a strong inverse correlation between vitamin D concentration in the blood and the COVID-19 infection. Patients with 25 (OH) D concentration below 20 ng/ml showed a 54% higher test positive index than those with 25 (OH) D blood values within the optimal range (>30 ng/ml). Moreover, vitamin D content has been proven to be independent of race, sex, age, latitude predictor of SARS-CoV-2 virus infection. This was also confirmed by the study of Ilie et al. (Ilie et al., 2020). They found a negative correlation between the average vitamin D concentrations in the blood in European patients inhabiting different latitudes and the number of COVID-19 cases per 1 million inhabitants.

There are several possible mechanisms by which vitamin D may reduce the risk of COVID-19 infection. They include, inter alia, the induction of the transcription of cathelicidin and defensin, which reduce the rate of virus replication and the promotion of macrophage chemotaxis to the site of inflammation (Kuźmińska 2012; Fiske et al., 2019).

Grant et al. (Grant et al., 2020) recommended that people at risk of COVID-19 consider vitamin D supplementation for several weeks at 10,000 IU/day to quickly replenish 25 (OH) D deficiency, followed by the 5,000 IU/day supplementation. The target vitamin D blood concentration should be in the range of 40–60 ng/ml. However, such recommendations to reduce the risk of infection with the SARS-CoV-2 virus still require well-designed, randomized, and placebo-controlled clinical trials.

### Vitamin D Role in Alleviating the Symptoms of SARS-CoV-2 Infections

Flu-like symptoms often characterize the early stage of COVID-19 disease. The meta-analysis by Zhu et al. (2020) shows that the most common symptoms of SARS-CoV-2 infection are fever (80.4%), fatigue (46%), and cough (63.1%). Other common symptoms are muscle soreness (33%), chest tightness (35.7%), and dyspnoea (35%). Existing data suggest that the severity of the disease caused by SARS-CoV-2 is mainly dependent on the individual immune response, which comorbidities may significantly impair.

Austrian prospective study by Pizzini et al. (Pizzini et al., 2020) analyzed the relationship of vitamin D with the clinical picture and the course of COVID-19. In this study, 109 patients infected with SARS-CoV-2 were enrolled and subjected to an 8-week detailed follow-up. Vitamin D deficiency has been shown to be common among patients. However, it appeared not to be a determinant factor for the disease severity and neither associated with changes in chest CT or abnormalities as revealed in lung function tests. Interestingly, it has been observed that people with severe disease show a disrupted parathyroid-vitamin D axis in the recovery phase. However, this may be associated with lower exposure to sunlight due to prolonged quarantine and hospitalization, or it may be a residual dysregulation following an infectious disease. Similar results are presented by Hernandez et al. (Hernandez et al., 2021). The study was designed as a case-control analysis involving 216 COVID-19 patients and 197 healthy volunteers. In the study group, vitamin D deficiency was found in 84% of patients, in the control group only in 47% (*p* < 0.0001). However, again, no relationship has been proven between vitamin D deficiency and the severity of the disease.

On the other hand, numerous studies suggest a protective role of vitamin D in potentially destructive changes appearing in lung tissue (Hansdottir and Monick, 2011; Xu et al., 2017). It has been proven that 25 (OH) D metabolites stimulate the production of surfactants in the alveoli (Rehan 2020). Perhaps this is why the protective effect of vitamin D in alleviating COVID-19 infection may be mainly noticeable in patients with severe symptoms of the infection and lung damage.

This hypothesis is confirmed by the results of the meta-analysis conducted by Pereira et al. (Pereira et al., 2020). They have observed that the vitamin D deficiency in COVID-19 patients was associated with a significantly higher risk of hospitalization and mortality. This time, a positive correlation has also been demonstrated between vitamin D concentration and the severity of symptoms.

Moreover, vitamin D may also alleviate the neurological symptoms associated with SARS-CoV-2 infection (Xu et al., 2020). The neuroprotective effect of vitamin D seems related to regulating the production of neurotrophins, which are factors determining the survival and differentiation of nerve cells (Di Somma et al., 2017). The SARS-CoV-2 virus, penetrating through the olfactory bulb, damages neurons leading to neurological symptoms such as pain and dizziness, confusion, and smell and taste disturbances (Favas et al., 2020). In experimental studies on animals, it was proved that vitamin D promotes the migration and proliferation of oligodendrocytes, enhancing the remyelination of damaged neurons (Gomez-Pinedo et al., 2020).

In COVID-19 patients hospitalized in Intensive Care Units, the most common are haemostatic disorders and hypercoagulability (43% of patients) related to these disorders (Pluta et al., 2021). Activation of the clotting pathways during the immune response to SARS-CoV2 infection contributes to organ hypoperfusion and multiorgan failure. These findings strongly suggest a protective effect of vitamin D on hemostatic disorders. The anticoagulant effect may be the result of an increase in glutathione and thrombomodulin and in the decrease in the concentration of tissue thromboplastin (blood coagulation factor III) (Vyas et al., 2020).

### Vitamin D in the Treatment of SARS-CoV-2 Infections

Currently, there is still insufficient scientific evidence to support that vitamin D supplementation may help treat COVID-19 infection. Most of the available studies are observational, so it cannot be clearly stated whether the lower vitamin D concentration is the cause or the consequence of the severe course of the disease. There is, however, a suggestion that adequate vitamin D supplementation in patients with COVID-19 may alleviate the course of the disease. Future well-designed clinical trials should verify this hypothesis.

Castillo et al. (2020) conducted a randomized, placebo-controlled clinical trial in 76 patients with viral pneumonia of SARS-CoV-2 etiology confirmed by radiological examination. Importantly, on admission, patients received hydroxychloroquine and azithromycin treatment. They were then randomly assigned to two groups - receiving an oral calcifediol (0.532 mg on the day of admission, and then 0.266 mg on the 3rd and 7th day of treatment) or placebo. It has been shown that COVID-19 patients treated with hydroxychloroquine and azithromycin who were additionally administered calcifediol required significantly less often the stay in the Intensive Care Unit as compared to the placebo-receiving group. In turn, (Murai et al., 2021) analyzed the effect of a single, high dose of vitamin D on the course of the treatment of patients with moderate and severe SARS-CoV-2 infection. The study was designed as a multicentre, randomized, and placebo-controlled study. It included 240 COVID-19 patients who were randomized to two groups: placebo (*n* = 120) and vitamin D 200,000 IU, as a single dose on admission (*n* = 120). It has not been observed that a single high dose of vitamin D, compared to a placebo, shortens the hospitalization time, reduces the risk of intensive care unit stay or the need for mechanical ventilation. Importantly, patients who were previously supplemented with vitamin D (at a dose >1000 IU/day) were excluded from this study.

In contrast to the above-described study, (Anweiler et al., 2020) designed a prospective clinical trial to evaluate the impact of regular vitamin D supplementation on the clinical course of patients infected with SARS-CoV2. This study enrolled 77 patients (mean age 88 ± 55) hospitalized due to COVID-19. The intervention groups included patients regularly supplemented with vitamin D during the previous year (Group 1) and people supplemented with vitamin D only after the diagnosis of COVID-19 (Group 2). The control group included participants who were not receiving vitamin D supplements (group 3). Then, the patients were monitored for 14 days. It has been shown that regular vitamin D3 supplementation was associated with less severe COVID-19 and a better survival rate in hospitalized patients. However, supplementation with 80,000 IU of vitamin D3 after diagnosis of COVID-19 did not improve the outcome of the COVID-19 treatments.

There are reports of the effectiveness of treatment with the vitamin D metabolite - calcifediol - in reducing mortality among patients with COVID-19 (Alcala Diaz et al. 2021). The study included 537 patients hospitalized due to SARS-CoV2 infection. The study group included patients who were administered calcifediol in the scheme: double dose on the day of admission, and then a single one on the 3rd, 7th, 14th, 21st, and 28th day of treatment. The control group consisted of patients who did not receive this treatment. It has been shown that patients from the study group achieved lower CURB-65 scores, assessing the severity of pneumonia. Acute respiratory distress syndrome was also significantly less common in them. A limitation of this study was the lack of randomization and placebo control.

Maghbooli et al. (2021) designed a randomized, double-blind, placebo-controlled study evaluating the effect of calcifediol treatment on the course of SARS-CoV-2 infection in hospitalized patients. The study included 106 patients diagnosed with vitamin D deficiency on the day of admission. Patients from the study group received calcifediol at a dose of 25 µg for 60 days, while patients from the control group received a placebo. A lower trend was observed in terms of hospitalization time in the Intensive Care Unit, ventilator therapy, and mortality in the group treated with calcifediol compared to the control group, but these differences were not statistically significant. On the other hand, there was a significant increase in the percentage of lymphocytes and a decrease in the neutrophil-lymphocytic index in patients from the study group, which correlated with a reduction in mortality during the first 30 days of treatment. Due to the relatively small sample size, the authors did not formulate any unequivocal recommendations regarding the use of calcifediol in the treatment of COVID-19.

Most of the results from published clinical trials indicate the potential benefits of vitamin D in treating SARS-CoV-2 infections (Table 2). However, some of them are still incomplete. Among those currently underway, a trend can be observed to improve the vitamin D supply pattern in COVID-19 (Table 3).

**TABLE 2:** Results of clinical trials on the role of vitamin D in the treatment of SARS-CoV2 infections.

No	Author (year)	Intervention	Dose of vitamin D	Results
1	Lakkireddy et al. (2021)	Daily supplementation of vitamin D for 8 or 10 days (depending upon BMI) or not	60,000 IU	Reduction of inflammatory markers: N/L ratio, CRP, LDH, IL6, Ferritin
2	Sabico et al. (2021)	Daily supplementation of two doses of vitamin D for 2 weeks	5,000 IU *vs.* 1,000 IU	Reduction the time to recovery for cough and gustatory sensory loss in group 5000 IU supplementation
3	Alcala-Diaz et al. (2021)	Treatment of calcifediol (double dose on entry and then one dose on day 3, 7, 14, 21, and 28) or not	0.266 mg calcifediol	Treatment with calcifediol was associated with lower in-hospital mortality
4	Maghbooli et al. (2021)	Treatment with calcifediol (one dose for 60 days) or placebo	25 μg calcifediol	Treatment with calcifediol was associated with increase in the lymphocyte percentage and decrease in the neutrophil-to-lymphocyte ratio
5	Elamir et al. (2021)	Treatment with calcitriol (one dose daily for 14 days or hospital discharge) or not	0.5 μg calcitriol	Improvement in oxygenation among patients treated with calcitriol
6	Murai Et al (2021)	Receive a single high dose of vitamin D3 or placebo	200,000 IU	Not significantly reduce hospital length of stay, in-hospital mortality, admission to the intensive care unit or need for mechanical ventilation
7	Castillo et al.(2020)	Treatment of calcifediol (double dose on entry and then one dose on day 3, 7) or placebo	0.266 mg calcifediol	Treatment with calcifediol was associated with less often the stay in the Intensive Care Unit

**TABLE 3:** Active clinical trials on the use of vitamin D in the treatment of SARS-CoV2 infections.

No	Title	Study type	Estimated enrollment	Intervention	Vitamin D dose	Estimated study completion Date
1	Efficacy of Treatment With Vitamin D in Patients Diagnosed With COVID-19 Who Presenting Vitamin D Deficiency and Pneumonia (Clinical Trial NCT04621058, 2021)	Randomized, Double-blind, Placebo-controlled Trial	108 participants	In case of Vitamin D concentration <30 or 40 ng/ml patients will take vitamin D supplements	0.266 mg or 0.532 mg	November, 2021
2	Low vs. Moderate to High Dose Vitamin D for Prevention of COVID-19 (Clinical Trial NCT04868903, 2021)	Randomized, Double-blind Trial	2000 participants	Half the subjects randomized to low dose vitamin D therapy (control group), and half to moderate or high (study group)	400 vs 4000 IU or 10 0000 IU	June, 2022
3	Phase 3 Randomised Controlled Trial of Vitamin D Supplementation to Reduce Risk and Severity of COVID-19 and Other Acute Respiratory Infections in the United Kingdom Population (Clinical Trial NCT04579640, 2021)	Randomized, open label clinical trial	6,200 participants	Subjects randomized to low dose vitamin D therapy (control group), and to moderate or high (study group)	400 *vs*. 800 IU or 3200 IU	June, 2021
4	A Cluster-Randomized, Double-Blind, Placebo-Controlled Study to Evaluate the Efficacy of Vitamin D3 Supplementation to Reduce Disease Severity in Persons With Newly Diagnosed COVID-19 Infection and to Prevent Infection in Household Members (Clinical Trial NCT04536298, 2021)	Randomized, Double-Blind, Placebo-Controlled Study	2,700 participants	Three capsules per day will be taken on days 1 and 2, and one capsule per day will be taken on days 3 through 28	1–2 day: 9600 IU 3–28 day: 3200 IU	December, 2021
5	Vitamin D Supplementation and Covid-19: a Randomised, Double- Blind, Controlled Study (Clinical Trial NCT04636086, 2021)	Randomised, Double- Blind, Placebo-Controlled Study	100 participants	One capsule per day will be taken on days: 1–4, 8, 15, 22, 29, 36	25 ,000 IU	31 January 2022

### Effect of COVID-19 Pandemic on Vitamin D Concentration Values in Patients

In the early days of the pandemic, many European countries introduced travel restrictions and a ban on leaving the domicile. These restrictions were intended to slow down the spread of the SARS-CoV-2 virus. One of the side effects of this quarantine was vitamin D deficiency. In a retrospective study conducted on a pediatric population in Poland (Rustecka et al., 2021), statistically significant lower concentration values of vitamin D were observed in children during the first year of the COVID-19 pandemic.

Similar results were obtained by (Yu et al., 2020), who analyzed the values of 25 (OH) D blood concentration in the population of children living in China. The authors of both studies indicate a reduced solar exposure and the resulting reduced skin synthesis of vitamin D as a potential cause of this phenomenon. Another interesting observation was the disturbance in the annual variability of vitamin D concentrations. During the pandemic period, the characteristic seasonal summer peak of 25 (OH) D values was not observed. Thus, the pandemic restrictions disrupt the natural sources of vitamin D and require specific supplementation.

## Conclusion

Overall, vitamin D has an immunomodulatory effect on the immune system. However, its impact on the risk of developing the disease and the course of SARS-CoV-2 infection is still not fully understood. Currently, no recommendations can be made regarding the role of vitamin D in treating COVID-19, while, definitely, its correct concentration value in the blood favors a less complicated course of the disease. Pandemic-related restrictions, mostly isolation and quarantine, impact vitamin D concentration value in the blood and thus may require supplementation.
